# Identification of Genetic Modifiers of TDP-43 Neurotoxicity in Drosophila

**DOI:** 10.1371/journal.pone.0057214

**Published:** 2013-02-27

**Authors:** Lihong Zhan, Keith A. Hanson, Sang Hwa Kim, Apeksha Tare, Randal S. Tibbetts

**Affiliations:** Department of Human Oncology, Program in Molecular and Cellular Pharmacology, University of Wisconsin School of Medicine and Public Health, Madison, Wisconsin, United States of America; King’s College London, United Kingdom

## Abstract

Cytosolic aggregation of the nuclear RNA-binding protein TDP-43 is a histopathologic signature of degenerating neurons in amyotrophic lateral sclerosis (ALS), and mutations in the *TARDBP* gene encoding TDP-43 cause dominantly inherited forms of this condition. To understand the relationship between TDP-43 misregulation and neurotoxicity, we and others have used Drosophila as a model system, in which overexpression of either wild-type TDP-43 or its ALS-associated mutants in neurons is sufficient to induce neurotoxicity, paralysis, and early death. Using microarrays, we have examined gene expression patterns that accompany TDP-43-induced neurotoxicity in the fly system. Constitutive expression of TDP-43 in the Drosophila compound eye elicited widespread gene expression changes, with strong upregulation of cell cycle regulatory genes and genes functioning in the Notch intercellular communication pathway. Inducible expression of TDP-43 specifically in neurons elicited significant expression differences in a more restricted set of genes. Genes that were upregulated in both paradigms included *SpindleB* and the Notch target *Hey*, which appeared to be a direct TDP-43 target. Mutations that diminished activity of Notch or disrupted the function of downstream Notch target genes extended the lifespan of TDP-43 transgenic flies, suggesting that Notch activation was deleterious in this model. Finally, we showed that mutation of the nucleoporin *Nup50* increased the lifespan of TDP-43 transgenic flies, suggesting that nuclear events contribute to TDP-43-dependent neurotoxicity. The combined findings identified pathways whose deregulation might contribute to TDP-43-induced neurotoxicity in Drosophila.

## Introduction

Amyotrophic lateral sclerosis (ALS) is an age-dependent, fatal motor neuron disease with no effective treatment. Approximately 90% of ALS arises sporadically (sALS) without clear genetic etiology, whereas 10% of ALS cases are familial (fALS) and have a clear genetic component. The first established genetic cause of ALS was dominant mutations of the *SOD1* gene, which was responsible for up to 20% of all fALS cases [1]. Well over 100 ALS-associated mutations in *SOD1* have been identified. Biochemically, ALS mutations reduced SOD1 solubility and promote its aggregation, leading to numerous toxic effects [2]. Mouse models of SOD1 have been a useful tool for elucidating ALS disease mechanisms. From these models it has emerged that SOD1-induced ALS was the result of its action in multiple cell types; for example, expression of SOD1 in astrocytes was deleterious for neighboring wild-type neurons [2]. However, the pathophysiologic mechanisms that contribute to ALS are still poorly understood.

Recent advances in ALS genetics promise to illuminate ALS disease pathways and have renewed hopes for a therapeutic breakthrough. Prominent among these advances was the discovery that dominant mutations in two functionally and structurally-related RNA-binding proteins, TDP-43 and FUS/TLS, caused familial forms of ALS [3], [4], [5], [6], [7], [8], [9], [10]. The identification of TDP-43 mutations was particularly striking given that cytosolic aggregation of TDP-43 was found to be a histopathologic signature of degenerating neurons in sALS and frontotemporal lobe degeneration (FTD) [11]. These discoveries have heralded a new era in ALS research in which altered RNA metabolism is viewed as a central element of the disease process [12]. Virtually all ALS-associated mutations in TDP-43 cluster in a C-terminal Gly-rich domain that has prion-like structural characteristics [13], [14]. ALS-associated mutations in this domain promote TDP-43 aggregation [14], [15]; however, the precise role of TDP-43 aggregation in disease initiation remains to be determined. FUS is also an aggregation-prone protein and disease associated mutations in a non-canonical PY-type nuclear localization sequence promote FUS cytosolic aggregation [16], [17], [18], [19].

Originally identified as a binding factor of the HIV transactivation region, TDP-43 is an essential gene that regulates mRNA splicing by virtue of binding to (UG)_n_ repeats in target RNAs [20]. TDP-43 mediates exon 9 skipping of the CFTR gene, which was its first identified RNA substrate [20]. Since its identification as an ALS gene, the RNA interactome of TDP-43 has been interrogated using next-generation sequencing approaches, which identified thousands of RNA substrates for TDP-43, including many with critical neuronal functions [21], [22], [23]. These studies revealed that TDP-43 preferentially binds to internal regions of long introns as well as 3′UTR sequences. Interestingly, TDP-43 also negatively regulates its own mRNA expression via direct binding to its 3′UTR [21], [24], [25]. It has been proposed that aggregation of TDP-43 disrupts this regulation, leading to the accumulation of TDP-43 mRNA, increased TDP-43 translation, and increased TDP-43 aggregation [21], [24]. This provocative feed-forward model for TDP-43 proteinopathy could contribute to the well-established phenomenon of ‘nuclear clearing’ in which the immunohistochemically detectable TDP-43 in the nucleus is severely diminished in affected brain regions of ALS and FTD-TDP patients [26].

Transgenic overexpression of wild-type or ALS mutants of TDP-43 induced robust neurotoxicity in transgenic animals, including mice, worms, and Drosophila (reviewed in [27]). Using a Drosophila model in which TDP-43 is selectively expressed in motor neurons (D42-Gal4/UAS-TDP-43 flies), we previously showed that TDP-43 promotes motor neuron degeneration in a dose-dependent and age-dependent manner, and similar findings were made by several other groups [28], [29], [30], [31], [32], [33], [34]. We further showed that the coexpression of the TDP-43-binding protein ubiquilin 1 (UBQLN1) [35] dramatically worsened TDP-43 associated phenotypes [34]. UBQLN1 contains dual ubiquitin-associated (UBA) and ubiquitin-like (UBL) domains and targets ubiquitylated substrates to one of two degradative fates in the cell: proteasome-dependent degradation or macroautophagy [36], [37], [38], [39]. Consistent with these functions, UBQLN1 promoted TDP-43 clearance in Drosophila neurons [34]; nonetheless, it exacerbated TDP-43 associated toxicity. The mechanisms whereby UBQLN enhances TDP-43 toxicity are not known.

The goal of the present study was to further characterize the process of TDP-43-dependent neurotoxicity in Drosophila, with emphasis on identifying associated RNA expression changes. We demonstrate upregulation of cell cycle and Notch pathways following constitutive TDP-43 expression. We also provide evidence that the Notch target gene *Hey* is a direct target of TDP-43 that is upregulated early in the course of TDP-43 induced neurotoxicity and that mutations in the Notch pathway extended lifespan in TDP-43 transgenic flies. These findings suggest that pathologic Notch activation contributes to neuronal abnormalities in this Drosophila model of ALS.

## Results

### Effect of Apoptosis Inhibition and Knockdown of *TBPH* on TDP-43 Phenotypes

**Figure 1:**
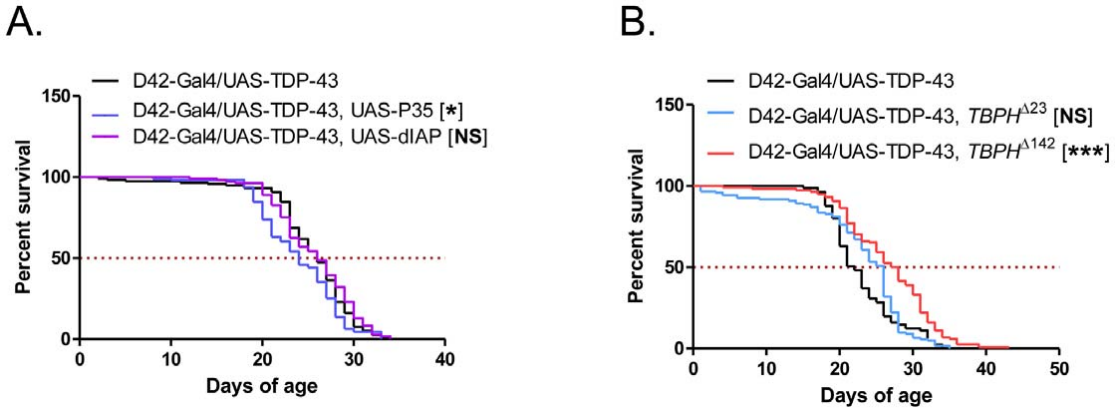
Effects of caspase inhibition and *TBPH* knockdown on D42-Gal4/UAS-TDP-43 fly lifespan. (**A**) Survival curves of D42-Gal4/UAS-TDP-43 flies crossed to UAS-P35 and UAS-dIAP lines. Median survival (days) of: D42-Gal4/UAS-TDP-43 = 26, N = 118; D42-Gal4/UAS-TDP-43, UAS-P35 = 24, N = 111, p<0.05; D42-Gal4/UAS-TDP-43, UAS-dIAP = 26, N = 109, p = 0.47. (B) Survival curves of D42-Gal4/UAS-TDP-43 flies crossed to two *TBPH* null lines. Median survival (days) of: D42-Gal4/UAS-TDP-43 = 22, N = 81; D42-Gal4/UAS-TDP-43, *TBPH* ^Δ23^ = 26, N = 122, p = 0.12; D42-Gal4/UAS-TDP-43, *TBPH* ^Δ142^ = 27.5, N = 118, p<0.0001.

Overexpression of human TDP-43 induced age and dose-dependent neurotoxicity in Drosophila [28], [29], [30], [31], [34], [40]. To assess whether apoptosis contributed to neurodegenerative phenotypes, we crossed flies expressing TDP-43 in motor neurons (D42-Gal4/UAS-TDP-43 flies) to two lines that express inhibitors of apoptosis, P35 and dIAP (Drosophila inhibitor of apoptosis). P35 is a viral protein that inhibits both autophagic and apoptotic cell death via broad inhibition of caspases, and dIAP is a closely related homolog [41]. Expression of both of these proteins inhibited neurotoxicity in a Drosophila model for the neurodegenerative disease, ataxia-telangiectasia (A-T) [42]. Since both proteins are under the control of the UAS promoter, they were only expressed in the same cells as TDP-43, in this case motor neurons. Surprisingly, expression of either P35 or dIAP did not rescue the shortened lifespan of D42-Gal4/UAS-TDP-43 flies (Fig. 1A). Thus, although TDP-43 induced degenerative changes in Drosophila photoreceptor neurons and motor neurons [34], this does not appear to occur through activation of classical programmed cell death pathways.

A recent study found that complete knockout of the Drosophila TDP-43 homolog, *TBPH*, leads to a reduction in lifespan very similar to that seen in our model [43]. In these flies, expression of human TDP-43 specifically in motor neurons rescued this phenotype, suggesting that human TDP-43 can compensate for the loss of the endogenous protein. One mechanism of TDP-43 toxicity in our model, therefore, is that the total nuclear dose of TBPH and TDP-43 exceeds a critical level leading to dysregulation of normal TBPH functions. In order to test this possibility, we crossed D42-Gal4/UAS-TDP-43 flies to two *TBPH* knockout strains previously described, *TBPH* ^Δ23^ and *TBPH* ^Δ142^ [43], leading to loss of one *TBPH* allele in the progeny. Ablating one copy of the endogenous *TBPH* caused a small increase in the lifespan of D42-Gal4/UAS-TDP-43 flies (Fig. 1B), though statistical significance was reached only for the TBPHΔ142 allele. The strength of rescue probably reflects the intrinsic difference in these alleles as previously described [43]. Our results suggest that TDP-43 toxicity is dependent on TBPH expression level, which argues that the total dose of TDP-43/TBPH is critical for neuronal function and dysregulation of TBPH targets and signaling may underlie TDP-43 phenotypes in vivo.

### Gene Expression Changes in Constitutive GMR-Gal4/UAS-TDP-43 Flies

Changes in gene expression can provide insights into the neurodegenerative process. In order to identify TDP-43-induced changes in gene expression *in vivo*, we crossed UAS-TDP-43 flies to GMR-Gal4 flies to drive expression of TDP-43 in the eye. Purified mRNA from 3–5-day old GMR-Gal4/UAS-TDP-43 and GMR-Gal4/+ control was analyzed using whole-genome microarrays. This analysis identified 511 genes whose expression changed >2 fold between GMR-TDP-43 and GMR-Gal4 flies at 95% confidence. Of these genes, 389 were upregulated and 212 were downregulated, and approximately 30% have annotated functions (Table S1). The top ten up- and downregulated annotated genes are shown in Table 1. Interestingly, the most highly upregulated and the most highly downregulated gene were both from the methuselah family, a group of G-protein coupled receptors (GPCRs) involved in the regulation of adult lifespan [44].

**Table 1:** Gene expression changes in TDP-43 flies.

Gene	Fold Change	Function
methuselah-like 6	149.708	GPCR
Twin of m4 (Tom)	58.974	Notch signaling
AdenylateCyclase A	38.963	cAMP generation
AdenylateCyclase E	31.417	cAMP generation
Ucp4b	20.388	uncouples oxidative phosphorylation
Tetraspanin 42Eb	16.764	transmembrane signaling
polo	15.890	protein kinase
Osiris 19	13.754	unknown
hedgehog	13.232	development
E(spl) mδ	10.154	Notch signaling
Osiris 2	0.327	unknown
lectin-28C	0.325	galactose binding
Larval serum protein 1β	0.296	nutrient transport
Larval serum protein 2	0.291	nutrient transport
Tetraspanin 42E	0.274	transmembrane signaling
Myo28B1	0.260	myosin ATPase
RabX5	0.175	small GTPase
robl37BC	0.174	microtubule transport
shadow	0.116	cytochrome activity
methuselah-like 8	0.014	GPCR

Top ten upregulated (top) and downregulated (bottom) genes identified via microarray analysis comparing GMR-Gal4/UAS-TDP-43 flies to GMR-Gal4/+ controls. Only annotated genes were included. Functions are from FlyBase, and include both experimentally derived functions and putative functions based on structure or homology.

We identified a number of functionally related groups of genes that were changed in our microarray (Table 2). One group of genes is involved in the regulation of mitochondria and oxidative processes in cells. These include *Ucp 4b* (uncoupling protein 4b), which is involved in uncoupling components of the electron transport chain to reduce oxidative phosphorylation, as well as cytochrome P450 homologs. Another group of genes are involved in regulation of the cell cycle. These include the mitotic kinases *polo* and *Cdc2* and the cell cycle regulatory phosphatase, *string/cdc25*, as well as multiple variants of spindle proteins and cyclins. Finally, a large number of genes upregulated in our microarray are targets of the Notch intercellular communication pathway. Notch signaling regulates neuronal differentiation; this pathway allows a signal-sending cell, fated to become a neuron, to prevent neuronal differentiation in adjacent target cells [45]. The signal-sending cell expresses the Notch ligands Delta and Serrate on its surface, which then binds to Notch receptors on the target cell, inducing cleavage of the Notch protein and release of the Notch intracellular domain (NICD). NICD then enters the nucleus, and in conjunction with other cofactors activates the transcription of target genes. These genes, such as those listed in Table 2, are themselves transcription factors that collectively prevent the differentiation of the target cell into a neuron.

**Table 2:** Gene expression changes in TDP-43 flies, arranged by category.

	Gene	Fold Change		Gene	Fold Change
	Ucp4B	20.387		polo	6.235
	Cytochrome P450-4g1	7.079		spindle E	5.968
	BthDselenoprotein	5.028		spindle B	4.763
**Mitochondrial/**	Cyp6a19	4.132		cdc2	4.761
**Redox**	Tim17a1	2.973		fizzy	4.096
	Cyp316a1	2.713	**Cell**	chickadee	4.037
	thioredoxin-2	2.679	**Cycle**	greatwall	3.911
				cyclin A	3.895
	Twin of m4 (Tom)	58.974		inscuteable	3.124
	E(spl) mδ	10.154		cyclin B3	3.055
	Hairy/E(spl)-related (Hey)	6.072		string	2.885
**Notch Target**	Brother of Bearded (BobA)	5.563			
**Genes**	E(spl) mγ	4.781			
	E(spl) m4	3.262			
	Bearded (Brd)	2.842			
	E(spl) mα	2.531			

All significantly changed Notch genes are shown, but only the most highly upregulated mitochondrial/redox and cell cycle genes are included.

**Figure 2:**
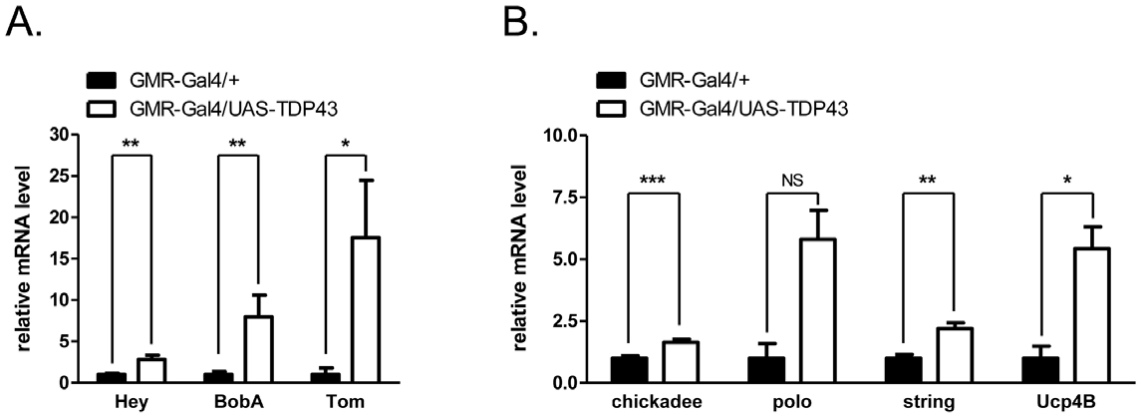
Validation of gene expression changes in TDP-43 flies. qPCR analysis of selected genes identified via microarray. (A) Upregulation of notch signaling genes by TDP-43 overexpression in the fly compound eye. (B) Changes of other genes by TDP-43 overexpression. GMR-Gal4/+ was treated as control sample. Data were presented as mean of four independent biological replicates with ±SEM.

Because aberrant Notch activation has been previously implicated in prion-induced neurotoxicity, we further investigated the cause and significance of Notch pathway upregulation in TDP-43 transgenic flies [46]. Validation by quantitative PCR assays revealed that the Notch target genes *BobA* (brother of bearded), *Tom* (Twin of M4), and *Hey* (Hairy/E(spl) related with YRPW motif) were strongly upregulated in whole heads of GMR-Gal4/UAS-TDP-43 flies versus GMR-Gal4/+ control (Fig. 2A). Quantitative PCR also validated the upregulation of *Ucp4b* and *string* in GMR-Gal4/UAS-TDP-43 flies (Fig. 2B). Finally, we also validated that the *Profilin* ortholog *chickadee*, was upregulated in GMR-Gal4/UAS-TDP-43 flies. Chickadee is a downstream RNA target of the Fragile X mental retardation (FMRP) protein that is implicated in axon pruning [47]. Interestingly, mutations in *Profilin1* gene were recently identified to cause familial ALS [48].

### Gene Expression Changes in Inducible TDP-43 GeneSwitch Flies

**Figure 3:**
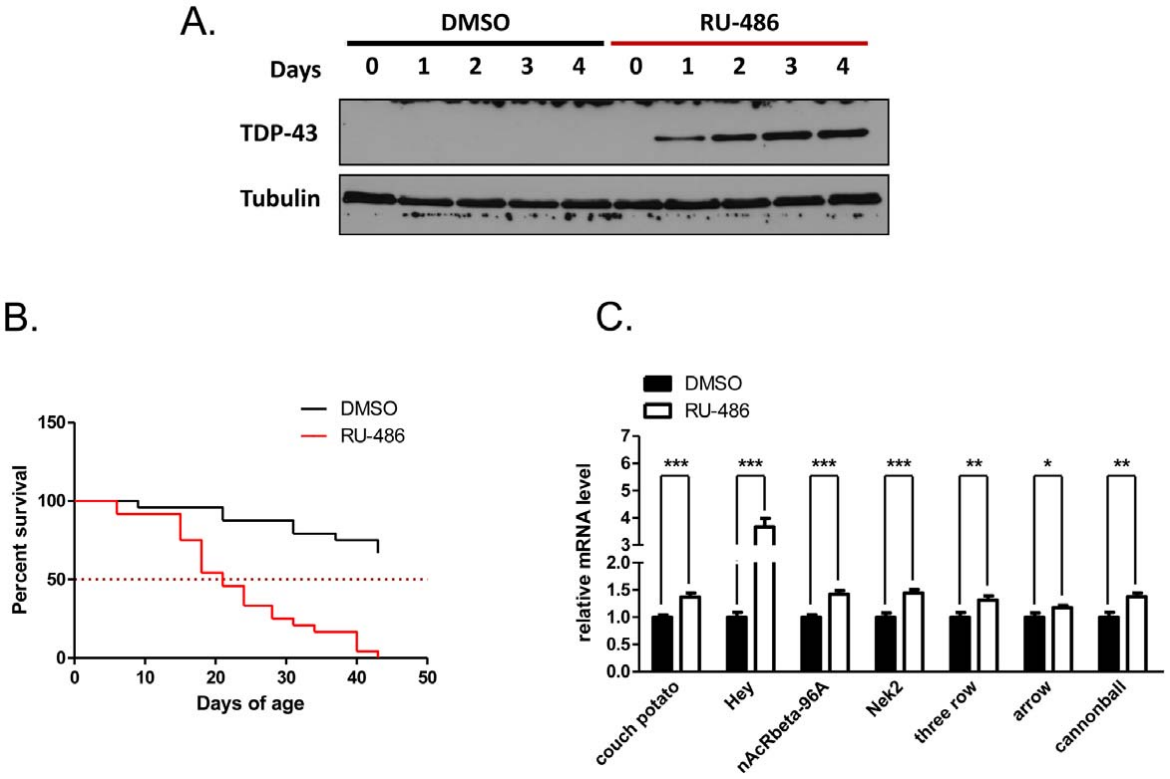
Inducible expression of TDP-43 in neurons *in vivo*. (**A**) Western blot analysis of TDP-43 expression in ELAV-GS-Gal4/UAS-TDP-43 flies treated with 1 mg/mL RU-486 or DMSO control over time. Loading was normalized to the equivalence of one fly per lane. Representative blot was shown. Tubulin was used as additional loading control. (**B**) Survival curves of ELAV-GS-Gal4/UAS-TDP-43 flies treated with 1 mg/mL RU-486 or DMSO control. N = 24 for each treatment, p<0.0001. (**C**) qPCR validation of inducible microarray hits. ELAV-GS-Gal4/UAS-TDP43 flies were treated with RU-486 for 3 days. Gene expression changes were compared to the DMSO treated control group. Data were presented as mean of four independent biological replicates with ±SEM.

Because GMR-Gal4 drives TDP-43 transgene constitutively beginning early in development, it is likely that many of the gene expression changes observed in this system were late events in TDP-43 pathogenesis and/or might be due to non-specific neurotoxicity. We therefore sought to identify gene expression changes that occur more directly due to TDP-43 expression using the Geneswitch (GS) inducible transgene expression system. In this approach, a fusion protein of Gal4 and the progesterone receptor (PR) is expressed only in neurons via the pan-neuronal ELAV promoter [49]. Upon addition of the PR ligand RU-486, the fusion protein is translocated to the nucleus and Gal4 is able to bind to UAS elements and activate transgene expression. We crossed UAS-TDP-43 flies to ELAV-GS flies and treated the progeny with RU-486 for varying period of time. As shown in Fig. 3A, TDP-43 expression was induced in a time-dependent manner, which peaked at day 3 and maintained at a steady level subsequently. Additionally, we performed a lifespan analysis comparing ELAV-GS-Gal4/UAS-TDP-43 flies given RU-486 to those given DMSO. The RU-486 treated flies had marked longevity loss (Fig. 3B) comparable to that observed using the constitutive D42-Gal4 driver, indicating that inducible expression of TDP-43 in adult neurons was sufficient to produce this phenotype. This result was similar to a previously published finding [32].

In order to identify the earliest TDP-43-induced gene expression changes, we compared ELAV-GS-Gal4/UAS-TDP-43 flies that had been treated with RU-486 for 3 days to those treated with DMSO over the same period. This time point was chosen because TDP-43 expression reached maximal by three days (Fig. 3A), just prior to the onset of the lifespan defect. Using the nominal 2-fold cutoff at 95% confidence, only seven annotated genes were differentially expressed in ELAV-GS-Gal4/UAS-TDP-43 flies upon RU-486 induction (Table 3). Three of these (*spindle B*, *white*, and the Notch target gene, *Hey*) were also upregulated in GMR-Gal4/UAS-TDP-43 flies. Overall, 63 genes were differentially expressed between ELAV-GS-Gal4/UAS-TDP-43 flies in the absence or presence RU-486 induction (any fold change, p<0.05). Forty-seven of these genes were upregulated in the presence of TDP-43 induction; 16 were downregulated. However, of the 63 differentially expressed genes only 22 had clearly annotated functions (Table S2). Table 4 shows genes identified in this analysis with known or potential roles in neuronal function that did not meet the strict 2-fold, p<0.05 cutoff. Validation of a subset of these genes confirmed that they were differentially expressed in the induced flies, in particular that the Notch target *Hey* was significantly upregulated after TDP-43 induction (Fig. 3C).

**Table 3:** Gene expression changes in inducible TDP-43 flies.

Gene	Fold Change	Function	Constitutive Array
white	3.399	ATPase/pigment	3.095
spindle B	3.369	meiosis	4.763
Hairy/E(spl)-related (Hey)	3.173	Notch signaling	6.072
CheB42b	0.451	chemosensation	0.552
Nuclear transport factor-2-related	0.431	nuclear import	0.927
osm-1	0.413	microtubule motor	0.828
insulin-like peptide 3	0.0658	receptor binding	0.991

All annotated genes identified by microarray comparison of ELAV-GS-Gal4/UAS-TDP-43 flies treated with RU-486 versus DMSO control are shown, using criteria of >2 fold change and p<0.05. Functions are from FlyBase, and include both experimentally derived functions and putative functions based on structure or homology.

**Table 4:** Expanded gene expression changes in inducible TDP-43 flies.

Gene	Fold Change	p value	Function	ConstitutiveArray
nACR beta 96A	1.794	0.0175	ion channel	0.980
Nek2	1.772	0.0304	kinase	5.283
arrow	1.519	0.0376	Wnt receptor	1.116
couch potato	1.991	0.0462	mRNA binding	0.802
three rows	1.523	0.0497	unknown	1.766
cannonball	2.541	0.0619	transcription	1.225

Genes are from same experiment and analysis as in Table 3, with less stringent criteria to identify potentially important genes. nACR, nicotinic acetycholine receptor.

The comparatively low number of genes identified using the inducible ELAV-GS-Gal4/UAS-TDP-43 paradigm suggests that most changes in GMR-Gal4/UAS-TDP-43 flies occur as an indirect response to TDP-43 misexpression and/or neurotoxicity. Given that the ELAV and GMR drivers direct TDP-43 expression in only partially overlapping cell types (e.g. photoreceptor neurons of the eye) it is also possible that the limited overlap reflects cell-type selective gene expression patterns following TDP-43 misexpression. The finding that *Hey* is upregulated in both systems suggests that Notch activation is a specific and perhaps direct response to TDP-43 misexpression in Drosophila.

### Role of Cell Cycle in TDP-43-dependent Neurotoxicity

**Figure 4:**
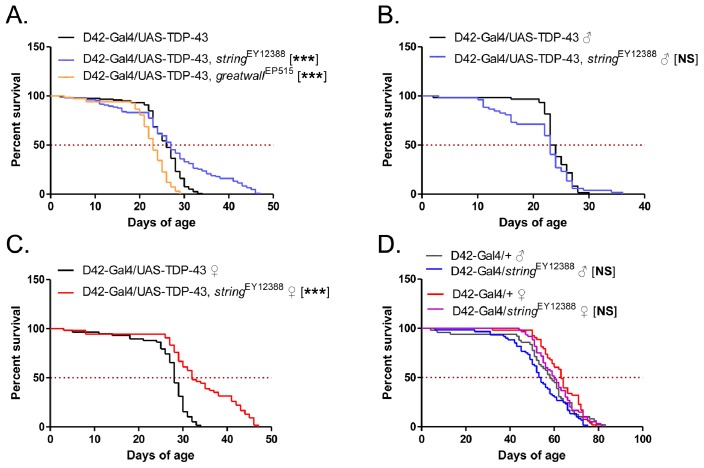
Effects of cell cycle gene mutations on D42-Gal4/UAS-TDP-43 fly lifespan. (**A**) Survival curves of D42-Gal4/UAS-TDP-43 flies in *string* and *greatwall* mutant backgrounds. Median survival (days) of: D42-Gal4/UAS-TDP-43 = 26, N = 118; D42-Gal4/UAS-TDP-43, *string* ^EY12388^ = 27, N = 106, p<0.0001; D42-Gal4/UAS-TDP-43, *greatwall* ^EP515^ = 23, N = 67, p<0.0001. (**B**) Effects of the *string* ^EY12388^ allele in males. Median survival (days) of: D42-Gal4/UAS-TDP-43 = 23.5, N = 60; D42-Gal4/UAS-TDP-43, *string* ^EY12388^ = 23, N = 52, p = 0.1831. (C) Effects of the *string* ^EY12388^ allele in females. Median survival (days) of: D42-Gal4/UAS-TDP-43 = 28, N = 58; D42-Gal4/UAS-TDP-43, *string* ^EY12388^ = 32, N = 54, p<0.0001. (**D**) Survival curves of D42-Gal4 control flies with the *string* ^EY12388^ mutant allele, divided by gender. Median survival (days) of males: D42-Gal4/+ = 58, N = 49; D42-Gal4/*string* ^EY12388^ = 53.5, N = 60, p = 0.08. Median survival (days) of females: D42-Gal4/+ = 63, N = 53; D42-Gal4/*string* ^EY12388^ = 60, N = 60, p = 0.43.

We next sought to determine whether differentially expressed genes contributed to TDP-43 neurotoxicity in Drosophila, focusing on cell cycle and Notch pathway genes. First, we examined two cell cycle genes: *string* and *greatwall*. *string* encodes the Drosophila homolog of mammalian Cdc25 family members, phosphatases that activate cyclin-dependent kinases during the cell cycle. *greatwall* encodes a kinase belonging to the protein kinase A, G, C (AGC) family that regulates chromatin condensation and G_2_/M cell cycle progression [50]. Though not the most upregulated cell cycle genes in our microarray, both of these genes were found to be suppressors of neurotoxicity in A-T flies [42]. We crossed D42-Gal4/UAS-TDP-43 flies to P-element insertion lines of both *string* and *greatwall*. While mutation of *greatwall* had no effect, mutation of *string* appears to extend the lifespan of a subset of D42-Gal4/UAS-TDP-43 flies (Fig. 4A). Interestingly, further analysis revealed that lifespan extension conferred by *string* mutation was restricted to female flies (Fig. 4B and C). In the absence of TDP-43, the *string* mutants exhibited slightly reduced lifespan (Fig. 4D), suggesting that the lifespan extension and gender difference may be specific to D42-Gal4/UAS-TDP-43 flies. These findings suggest that cell cycle suppression could partially reduce neurotoxicity of TDP-43.

### Notch Pathway Mutations Extend Lifespan of D42-Gal4/UAS-TDP-43 Flies

**Figure 5:**
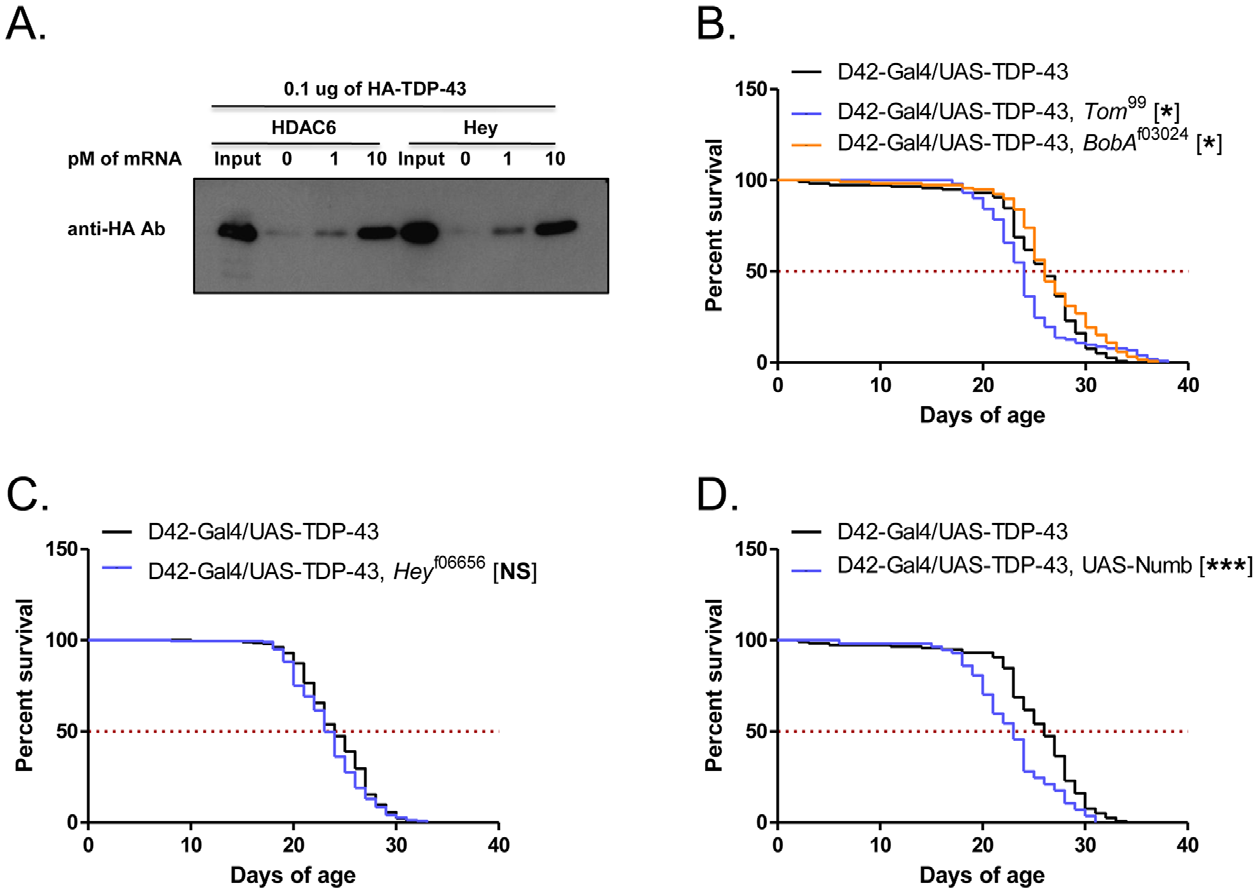
Binding of TDP-43 to Hey mRNA and effects of Notch pathway mutations on D42-Gal4/UAS-TDP-43 fly lifespan. (**A**) Immunopurified HA-TDP-43 was incubated with increasing amounts of Hey pre-mRNA that was attached to the biotin-streptavidin beads. The captured HA-TDP-43 was eluted and examined by western-blot. HDAC6 was included as positive control for the assay. (**B**) Survival curves of D42-Gal4/UAS-TDP-43 flies crossed to the mutant allele *Tom* ^99^ and *BobA* ^f03024^. Median survival (days) of: D42-Gal4/UAS-TDP-43 = 26, N = 118; D42-Gal4/UAS-TDP-43, *Tom* ^99^ = 24, N = 102, p<0.05; D42-Gal4/UAS-TDP-43, *BobA* ^f03024^ = 26, N = 119, p<0.05. (**C**) Survival curves of D42-Gal4/UAS-TDP-43 flies crossed to the mutant allele and *Hey* ^f06656^. Median survival (days) of: D42-Gal4/UAS-TDP-43 = 24, N = 213; D42-Gal4/UAS-TDP-43, *Hey* ^f06656^ = 23, N = 221, p = 0.0843. (**D**) Survival curves of D42-Gal4/UAS-TDP-43 flies crossed to overexpression line UAS-Numb. Median survival (days) of: D42-Gal4/UAS-TDP-43 = 26, N = 118; D42-Gal4/UAS-TDP-43, UAS-Numb = 23, N = 57, p<0.0001.

Aberrant activation of Notch is believed to contribute to the neuropathogenesis of stroke and prion disorders and antagonizes axon regeneration [46], [51], [52]. Furthermore, the Notch target genes upregulated in our array represent a significant percentage of known Notch targets. Most Notch target genes were found in two major genomic loci: the Enhancer of Split (E(spl)) locus and the Bearded (Brd) complex. Our array identified 4/13 genes in the E(spl) complex (*mδ, mγ, m4,* and *mα*) and 3/6 genes in the Brd complex (*Tom*, *BobA*, *Brd*). Additionally, the Notch target gene *Hey*, which is not found in either genetic complex, was identified in both the GMR-Gal4/UAS-TDP-43 constitutive and the ELAV-GS-Gal4/UAS-TDP-43 inducible array, suggesting that this gene could be a direct target of TDP-43-dependent regulation. Consistent with this idea, we found that TDP-43 bound to *in vitro*-transcribed Hey RNA (Fig. 5A) to an extent comparable to that observed for HDAC6, a known TDP-43 target substrate [53].

**Figure 6:**
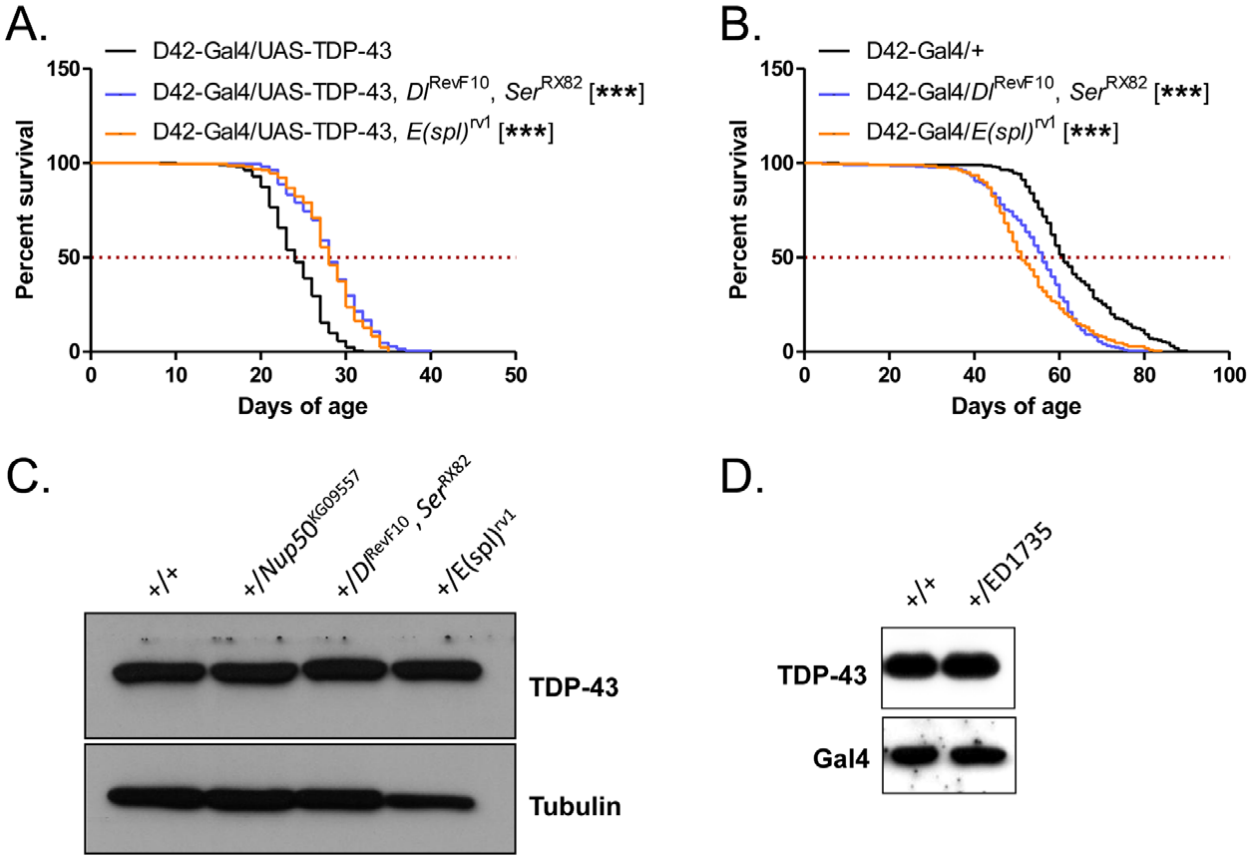
Effects of Notch pathway knockdown on D42-Gal4/UAS-TDP-43 and control fly lifespan. (**A**) Survival curves of D42-Gal4/UAS-TDP-43 flies crossed to mutants of *Delta* (*Dl* ^RevF10^) and *Serrate* (*Ser* ^Rx82^) or *Enhancer of split* (*E(spl)* ^rv1^). Median survival (days) of: D42-Gal4/UAS-TDP-43 = 24, N = 213; D42-Gal4/UAS-TDP-43, *Dl* ^RevF10^ *Ser* ^RX82^ = 28, N = 215, p<0.0001; D42-Gal4/UAS-TDP-43, *E(spl)* ^rv1^ = 28, N = 220, p<0.0001. (**B**) Survival curves of D42-Gal4 control flies containing *Dl* ^RevF10^ *Ser* ^Rx82^ and *E(spl)* ^rv1^ LOF alleles. Median survival (days) of: D42-Gal4/+ = 61, N = 221; D42-Gal4/*Dl* ^RevF10^, *Ser* ^RX82^ = 56, N = 219, p<0.0001; D42-Gal4/*E(spl)* ^rv1^ = 51, N = 216, p<0.0001; (**C** and **D**) TDP-43 expression level was not affected by the indicated mutations. D42-Gal4/UAS-TDP-43 flies were crossed to *w*1118 control flies or to the *Dl* ^RevF10^ *Ser* ^Rx82^, *E(spl)* ^rv1^, *Nup50* ^kg09557^, ED1735 deletion line. Flies containing D42-Gal4/UAS-TDP-43 were heterozygous with each testing allele. Tubulin or Gal4 was used as a loading control; blots were representative of multiple experiments.

To assess the contributions of *Hey* and other upregulated Notch targets to TDP-43-dependent lifespan reduction, we crossed D42-Gal4/UAS-TDP-43 flies to P-element insertion lines of *BobA* and *Hey*, and a deletion line of *Tom*. In all three cases, no effect was seen on the lifespan of D42-Gal4/UAS-TDP-43 flies (Fig. 5B and C). Additionally, we overexpressed the protein Numb in motor neurons in addition to TDP-43. Numb is a key cell fate determinant that antagonizes Notch; however, overexpression of Numb similarly did not rescue the D42-Gal4/UAS-TDP-43 phenotype (Fig. 5D). We hypothesized that since a large number of Notch target genes were changed in TDP-43 flies, knockdown of single genes may not be sufficient to affect this phenotype. Therefore, we crossed D42-Gal4/UAS-TDP-43 flies to a line harboring deletions in both *Delta* and *Serrate*, *Dl* ^RevF10^ *Ser* ^Rx82^ [54], the predominant Notch ligands. Crossing to this line significantly (p<0.0001) increased the lifespan of D42-Gal4/UAS-TDP-43 flies by 16% in median survival (Fig. 6A), but not control D42-Gal4/+ flies (Fig. 6B), suggesting that knockdown of Notch signaling may rescue TDP-43 phenotypes. We also crossed D42-Gal4/UAS-TDP-43 flies to flies harboring a chromosomal inversion leading to loss of the entire *E(spl)* complex, *E(spl)* ^rv1^ [55], and observed a similar extension of lifespan (Fig. 6A and B). Using Western blotting we confirmed that these mutations did not affect TDP-43 protein expression (Fig. 6C). These findings support a deleterious role for Notch pathway activation in TDP-43 induced neurotoxicity. Finally, although monoallelic *Hey* mutations did not rescue shortened lifespan in D42-Gal4/UAS-TDP-43 flies, overexpression of *Hey* specifically in motor neurons, independent of TDP-43, was embryonic lethal (data not shown and Ref. [56]). We have previously reported that high expression of TDP-43 in motor neurons is also embryonic lethal [34]. It is tempting to speculate that upregulation of Hey contributes to TDP-43-associated embryonic lethality.

### A mutation in the Nucleoporin Nup50 Strongly Enhanced Lifespan of D42-Gal4/UAS-TDP-43 Flies

**Figure 7:**
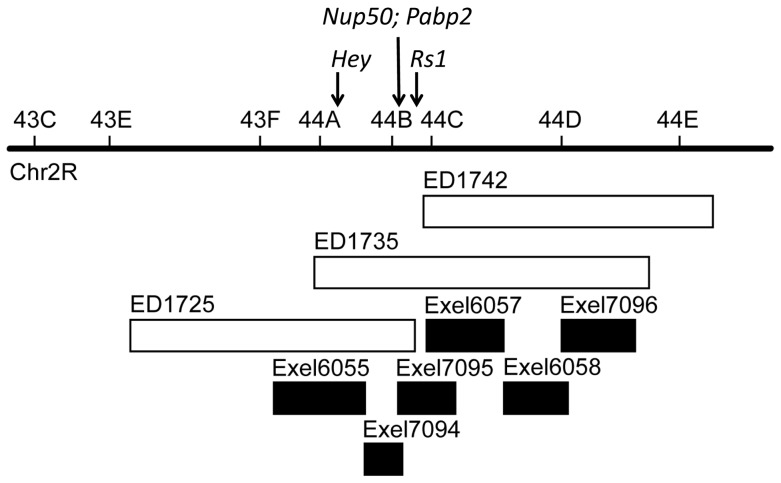
Genomic map of chromosomal deficiencies around the *Hey* locus. Deficiency lines used in Figure 8 and the location of genes tested in Figure 9 were as indicated. White boxes represented DrosDel collection while black boxes, Exelixis collection. Genomic mapping was adapted from Flybase GBrowse program (http://flybase.org/cgi-bin/gbrowse/dmel/).

**Figure 8:**
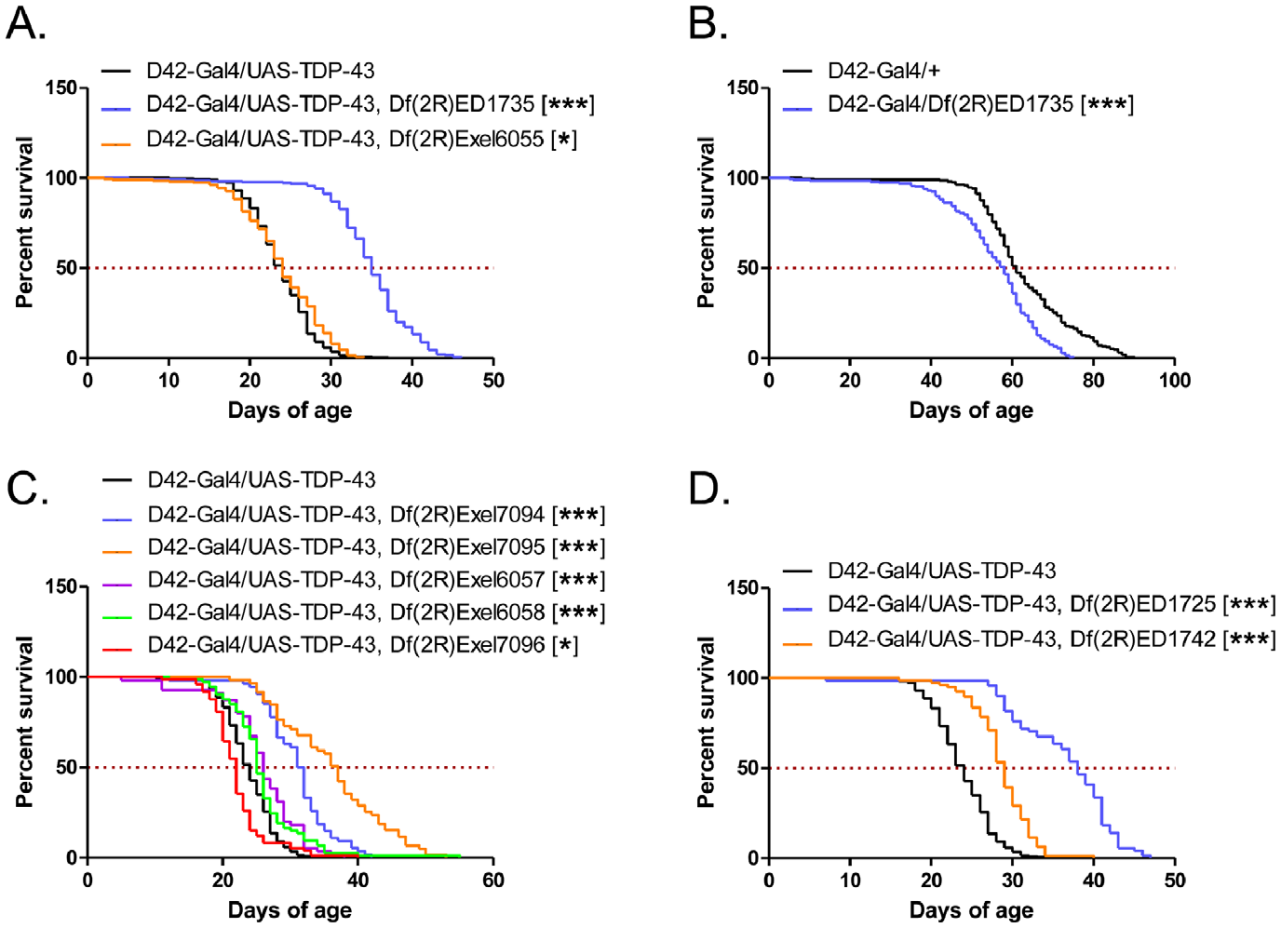
Analysis of chromosomal deficiency lines. (**A**) Survival curves of D42-Gal4/UAS-TDP-43 flies crossed to the Exel6055 and ED1735 deficiency lines. Median survival (days) of: D42-Gal4/UAS-TDP-43 = 24, N = 285; D42-Gal4/UAS-TDP-43, Df(2R)ED1735 = 35, N = 238, p<0.0001; D42-Gal4/UAS-TDP-43, Df(2R)Exel6055 = 24, N = 215, p<0.05. (**B**) Survival curves of D42-Gal4 control flies crossed to the ED1735 deficiency line. Median survival (days) of: D42-Gal4/+ = 61, N = 226; D42-Gal4/Df(2R)ED1735 = 58, N = 221, p<0.0001. (**C**) Survival curves of D42-Gal4/UAS-TDP-43 flies crossed to sub-deletions from the Exelixis collection. Median survival (days) of: D42-Gal4/UAS-TDP-43 = 24, N = 285; D42-Gal4/UAS-TDP-43, Df(2R)Exel7094 = 31.5, N = 54, p<0.0001; D42-Gal4/UAS-TDP-43, Df(2R)Exel7095 = 37, N = 59, p<0.0001; D42-Gal4/UAS-TDP-43, Df(2R)Exel6057 = 26, N = 55, p<0.0001;D42-Gal4/UAS-TDP-43, Df(2R)Exel6058 = 25, N = 73, p<0.0001; D42-Gal4/UAS-TDP-43, Df(2R)Exel7096 = 22, N = 73, p<0.05. (D) Survival curves of D42-Gal4/UAS-TDP-43 flies crossed to the DrosDel lines. Median survival (days) of: D42-Gal4/UAS-TDP-43 = 24, N = 285; D42-Gal4/UAS-TDP-43, Df(2R)ED1725 = 38, N = 71, p<0.0001; D42-Gal4/UAS-TDP-43, Df(2R)ED1742 = 29, N = 79, p<0.0001. All deletions are heterozygous on the second chromosome.

During the course of our studies of *Hey* as a possible TDP-43 modifier gene, we identified a deletion on the second chromosome from the DrosDel collection (ED1735) that substantially increased the lifespan of D42-Gal4/UAS-TDP-43 flies, while an adjacent and partially overlapping deletion from the Exelixis collection (Exel6055) had no effect, even though both spanned the *Hey* locus (Fig. 7 and Fig. 8A). The ED1735 deletion did not increase the lifespan of control D42-Gal4/+ flies (Fig. 8B), suggesting that a gene or genes in the affected region may be specifically modifying TDP-43 toxicity. Furthermore, TDP-43 and Gal4 protein levels were not changed by this deletion (Fig. 6D), ruling out changes in TDP-43 protein dose as a cause of the rescue.

ED1735 contains ∼100 genes, any of which could be responsible for the observed phenotype. We therefore tested sub-deletions from this genomic region in order to narrow down the gene list. We crossed D42-Gal4/UAS-TDP-43 flies to five deficiency lines from the Exelixis collection (Fig. 8C) and two from the DrosDel collection (Fig. 8D). The Exelixis lines generally delete a smaller genomic region and are therefore more useful in identifying candidate genes. Two of these lines, Exel7094 and Exel7095, rescued TDP-43 toxicity similar to the original ED1735 line, though two of the other lines also had small effects. Both lines from the DrosDel collection, which represent non-overlapping genomic regions, also rescued TDP-43 toxicity, suggesting that multiple genes in this region may be important.

Given these results, we focused on the Exel7094 and Exel7095 lines, which contain 17 and 18 genes, respectively. Interestingly, these deletions partially overlap, with six genes in common; the other strong rescuing line, ED1725, also contains this overlap region, further implicating these genes. These six candidate genes are: *Nup50* (Nucleoporin 50), *Coilin*, *Socs44a* (Suppressor of Cytokine Signaling at 44a), *CG42516*, *Pbp49* (PSEA-binding protein of 49 kDa), and *Pabp2* (poly-A binding protein 2). Two of the genes have potential direct links to TDP-43. *Nup50* encodes a nuclear pore protein, which may be involved in the nuclear import and export of TDP-43. The mammalian homolog of Pabp2 is known to be in a complex with TDP-43 where they potentially coregulate mRNA [57]. We therefore tested a P-element *Nup50* line and two P-element *Pabp2* lines for modification of D42-Gal4/UAS-TDP-43 fly lifespan. Additionally, we also tested a P-element line for the gene *Rs1*, which is within the deletion region of Exel7095 just adjacent to this overlap region and encodes an RNA helicase.

**Figure 9:**
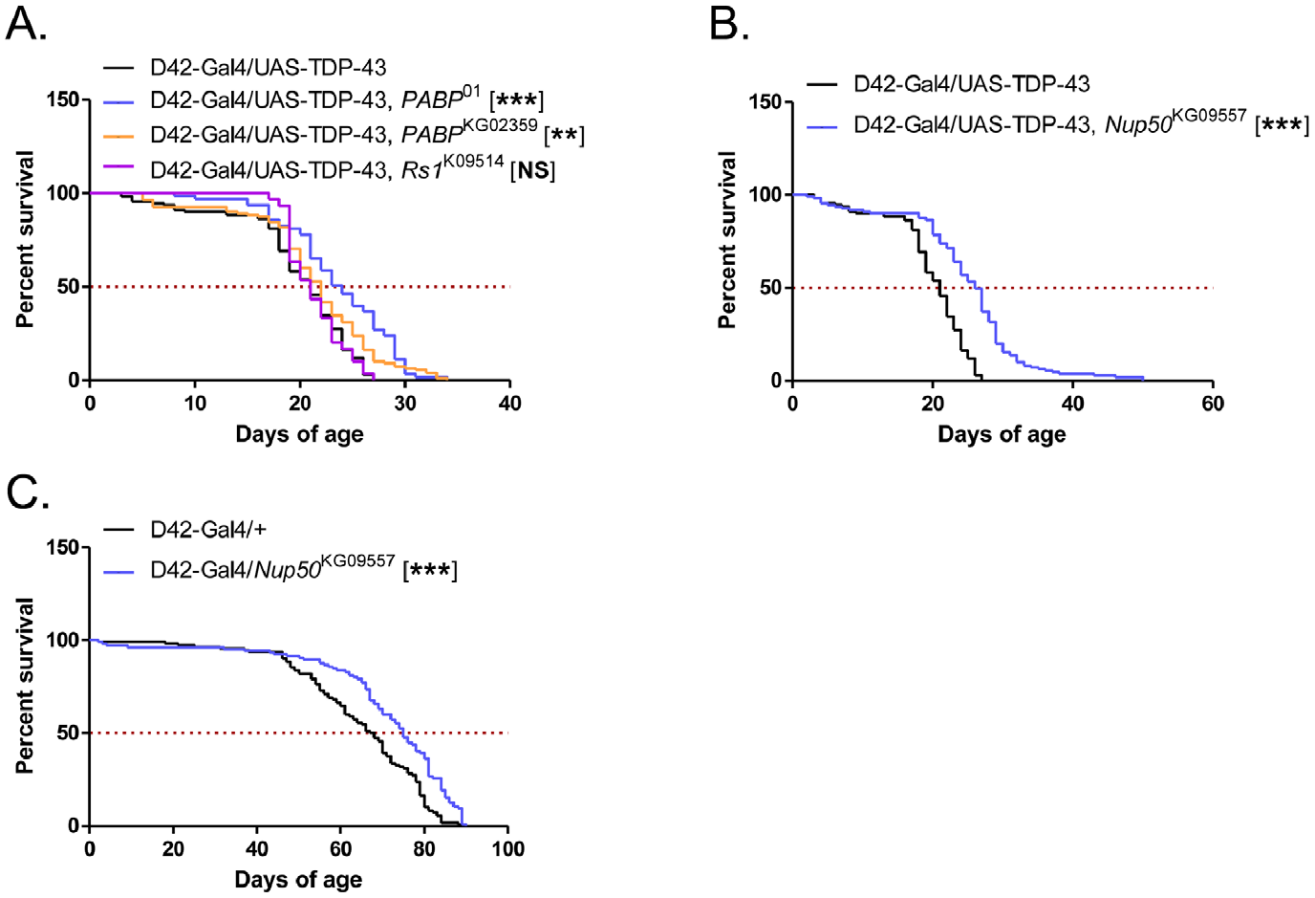
Analysis of *Nup50* and other candidate genes. (**A**) Survival curves of D42-Gal4/UAS-TDP-43 crossed to *Pabp2* (*Pabp2* ^01^or *Pabp2* ^kg02359^) and the *Rs1* ^k09514^ mutant lines. Median survival (days) of: D42-Gal4/UAS-TDP-43 = 21, N = 110; D42-Gal4/UAS-TDP-43, *PABP* ^01^ = 24, N = 63, p<0.0001; D42-Gal4/UAS-TDP-43, *PABP* ^KG02359^ = 22, N = 110, p<0.01. D42-Gal4/UAS-TDP-43, *Rs1* ^K09514^ = 21, N = 30, p = 0.8. (**B**) Survival curves of D42-Gal4/UAS-TDP-43 flies with the *Nup50* ^kg09557^ mutation. Median survival (days) of: D42-Gal4/UAS-TDP-43 = 21, N = 110; D42-Gal4/UAS-TDP-43, *Nup50* ^KG09557^ = 26, N = 111; *p*<0.0001. (**C**) Survival curves of D42-Gal4 control flies with *Nup50* ^kg09557^ mutation. Median survival (days) of: D42-Gal4/+ = 67.5, N = 110; D42-Gal4/*Nup50* ^KG09557^ = 75, N = 105, p<0.0001.

As shown in Fig. 9A, *Pabp2* and *Rs1* mutation had little or no effect on D42-Gal4/UAS-TDP-43 fly lifespan. However, *Nup50* mutation significantly rescued D42-Gal4/UAS-TDP-43 flies with 23% increase in median survival (Fig. 9B). Interestingly, the *Nup50* mutation seemed to preferentially increase maximum lifespan of D42-TDP-43 flies. This result suggests that *Nup50* is at least partially responsible for the rescue seen with deletion line ED1735; however, because the phenotypic rescue with *Nup50* mutation is not as large as that seen with the ED1735 deficiency line, other genes in this region may also be modifying TDP-43 toxicity. Additionally, unlike the ED1735 deletion, we found that *Nup50* mutation also moderately impacted the lifespan of control D42-Gal4 flies with 11% increase in the median survival (Fig. 9C). This suggests that part of the rescue seen with Nup50 may be non-specific. Finally, we found that this rescue was not due to changes in TDP-43 expression level (Fig. 6C).

**Figure 10:**
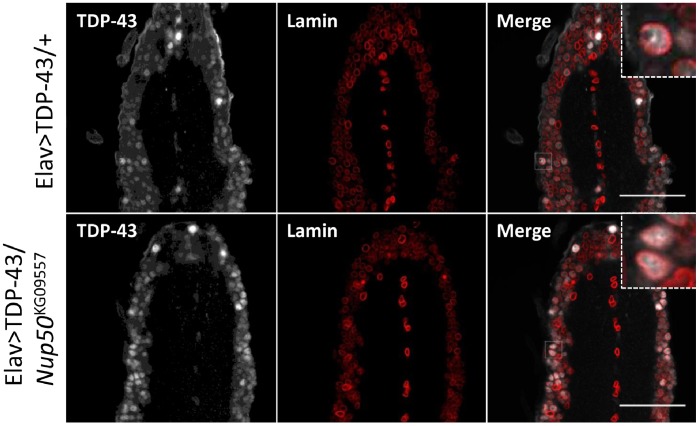
TDP-43 localization in the *Nup50* mutant background. Elav-Gal4/UAS-TDP-43 flies were crossed to either control (top panels) or *Nup50* ^kg09557^ (bottom panels) flies. Ventral nerve cords from 3^rd^ instar wandering larvae were dissected and immunostained for TDP-43 and the nuclear marker lamin. Scale bar indicated 50 um distance.

We recently showed that mutations in the Type 1 inositol triphosphate receptor (ITPR1) mitigated TDP-43-induced neuron dysfunction and caused TDP-43 to accumulate in the cytoplasm of Drosophila neurons [58]. To test whether *Nup50* mutations altered the nucleocytoplasmic distribution of TDP-43, we performed confocal microscopy of TDP-43 in larval brains of ELAV-Gal4/UAS-TDP-43 flies crossed to the *Nup50* mutant line. TDP-43 retained a predominantly nuclear localization pattern in both control and *Nup50* mutant larvae (Fig. 10). Although a subtle effect could not be completely ruled out, these results suggest that *Nup50* mutation does not extend lifespan by grossly altering the nucleocytoplasmic distribution of TDP-43.

## Discussion

Overexpression of wild-type or mutant TDP-43 proteins induces neurotoxicity in all metazoan models tested when a critical threshold of neuronal expression is surpassed [27]. The total level of TDP-43/TBPH activity seems to be a critical determinant of neurotoxicity, as deletion of *TBPH* extends D42-Gal4/UAS-TDP-43 fly lifespan (Fig 1B). Our studies support the general idea that neurons must maintain a relatively narrow range of TDP-43 expression and that disruption of TDP-43 autoregulatory mechanisms is likely to instigate the neurodegenerative process [26]. Interestingly, TDP-43 induced neuron dysfunction does not appear to involve caspase activation, which is similar to results observed upon misexpression of polyglutamine-expanded Huntingtin fragment [59]. Thus, although degenerative changes are observed in both photoreceptor neurons and motor neurons in TDP-43 transgenic Drosophila [15], [30], [34], paralysis and death in these flies may not be attributable to neuron death *per se*, but rather a consequence of severely perturbed neuronal function secondary to wholesale changes in gene regulation. This is consistent with reports in the literature that TDP-43 regulates the *Drosophila* neuromuscular junction [40].

Our study is one of several recent reports examining the impact of TDP-43 misexpression on neuronal gene expression and RNA splicing. Microarray analysis revealed hundreds of gene expression changes in GMR-Gal4/UAS-TDP-43 flies as compared to controls. A number of these genes are functionally related and are implicated in neurodegenerative processes. Mitochondrial and redox genes such as *Ucp4b* and the cytochrome P450 homologs contribute to cellular oxidative homeostasis, disruption of which has been implicated in neurodegenerative disease, including SOD1 ALS [60]. Additionally, we found numerous cell cycle regulatory genes upregulated in TDP-43 flies. Some of these genes, such as *string* and *greatwall*, were also found to be altered in A-T flies, and mutations of these two genes rescued A-T phenotypes [42]. We similarly found that *string* mutation rescued our TDP-43-induced lifespan defect, though for unknown reasons only in female flies. These findings suggest that cell cycle activation and progression in neurons may be important in the pathogenesis of ALS and other neurodegenerative diseases, as put forward by others [61].

Additionally, we found that TDP-43 upregulated a number of Notch target genes, implying activation of this cell differentiation pathway *in vivo*. Notch activation has previously been implicated in prion disease [46], suggesting a potential common mechanism with ALS; intriguingly, the C-terminal domain of TDP-43 has prion-like characteristics. Although knockdown of individual Notch target genes did not rescue the TDP-43 lifespan phenotype, global blockade of Notch signaling, via simultaneous deletion of *Delta/Serrate* and inversion of the entire *E(spl)* locus, did significantly rescue the lifespan of TDP-43 flies, suggesting that aberrant activation of Notch is contributing to neuronal dysfunction in these flies. This idea is further supported by the fact that at least one Notch gene studied, *Hey*, is upregulated within 72 h of TDP-43 overexpression in the ELAV-GS-Gal4/UAS-TDP-43 system, though monoallelelic mutation of *Hey* was insufficient to rescue neurotoxicity in D42-Gal4/UAS-TDP-43 flies. Interestingly, Hey appears to be a direct RNA target of TDP-43 (Fig. 5A) and we presume that many other differentially expressed genes are as well. For instance, couch potato (*cpo*), which was overexpressed in ELAV-GS-Gal4/UAS-TDP-43 flies, harbors an exceptionally large intron with an extensive TG repeat that is a strong candidate for binding by TDP-43 [21], [22].

The mechanism by which Notch activation contributes to TDP-43 toxicity is unknown. We speculate that upregulation of Notch–occurring due to direct TDP-43 RNA-binding activity or in response to TDP-43-mediated neuronal injury–inhibits axonal regrowth at compromised neuromuscular junctions in TDP-43 transgenic flies. Although further work is needed to address whether Notch activation contributes to axonal defects in TDP-43 transgenic flies, this model is compatible with recent findings showing that Notch inhibits regrowth of laser-transected axons in *C. elegans* [52].

Hazelett et al. recently reported gene expression changes in *Drosophila* with overexpression and knockout of TBPH [62]. A few notable genes were identified in our analysis as well as theirs; they found that *arrow* expression (Table 4) was changed with TBPH overexpression, and that the expression of multiple tetraspanins (Table 2) and acetylcholine receptors (Table 4) was changed with TBPH knockdown. However, the majority of genes we identified were not found in their analysis; interestingly, even within their study there was not significant overlap between genes whose expression changed with TBPH overexpression as compared to its knockout. Such discrepancy may be a result of different Gal4 driver lines used in both studies, which may reflect tissue specific regulation of TDP-43 targets.

Our finding that cell cycle and Notch targets are deregulated in TDP-43 flies is at least partially supported by findings from mammalian cells. A number of studies have identified TDP-43 target genes that regulate the cell cycle [22] or neuronal differentiation, notably including mouse Notch1 [63], human Notch3 [64], and the *Hey* mouse ortholog Hes5 [63]. However, these studies as well as ours indicate that a large percentage of the genome is regulated by TDP-43. It is therefore unlikely that there is a single gene or small group of genes that mediate TDP-43 toxicity in Drosophila; rather, activation of multiple pathways and processes are necessary for motor neuron dysregulation and degeneration. Thus, targeting of pathways rather than individual genes may be a better approach for identifying future ALS therapies.

Finally, during the course of this study we serendipitously discovered that a *Nup50* mutation increased the lifespan of D42-Gal4/UAS-TDP-43 flies. The role for *Nup50* was supported by multiple overlapping deletion mutants and a *Nup50* P-element insertion allele. Although the mechanism whereby *Nup50* mutation extends lifespan of D42-Gal4/UAS-TDP-43 flies is unknown, the most intriguing possibility is that *Nup50* mutation reduces TDP-43 nuclear import, which is thought to be important for its toxicity in the fly system [32]. However, unlike mutations in the Drosophila ITPR1 receptor, which diminish TDP-43 nuclear accumulation and extend longevity [58], *Nup50* mutation did not grossly affect TDP-43 localization in fly larval neurons (Fig. 10). Nevertheless, it is possible that *Nup50* mutation altered TDP-43 nuclear localization below the limits of detection using this qualitative assay. Alternatively, *Nup50* mutation may disrupt the nuclear import of proteins that mediate TDP-43 neurotoxicity. It is intriguing that *Nup50* mutation also increased the lifespan of control flies; however, we found that there was an increase in maximum lifespan that appears to be unique to TDP-43 flies. The importance of this observation remains to be seen, and further work will be needed to identify the exact molecular mechanism underlying the genetic interactions between *Nup50*, the Notch pathway, and TDP-43 *in vivo*.

## Materials and Methods

### Drosophila Maintenance

Flies were maintained with the standard cornmeal-molasses-yeast medium and all crosses were performed at 25°C. To facilitate the study of genetic modification using TDP-43 transgenic fly model, we generated a recombinant line in which UAS-TDP-43^ L2^ [34] and D42-Gal4 were both balanced on the same chromosome. For all experiments performed, flies were heterozygous for the D42-Gal4, UAS-TDP-43, and the indicated testing allele(s). For GeneSwitch inducible studies, ELAV-GS-Gal4/UAS-TDP-43 flies were treated with 1 mg/mL RU-486 dissolved in DMSO. RU-486 containing food was prepared by administering RU-486 to the fly food surface 24 hr prior to use. RU-486 treated flies were maintained in the drug containing food until specified harvesting time point.

### Drosophila Stocks

The *w*1118 strain was used as wild-type control in this report. These stocks were generously provided by: Dr. Feiguin (*TBPH* ^Δ23^; *TBPH* ^Δ142^; [43]) Dr. John Tower (ELAV-GS; [65]); Dr. Manfred Frasch (*Tom* deletion; [66]); Dr. Bingwei Lu (UAS-Numb; [67]); and Dr. Christos Delidakis (UAS-Hey; [56]). The UAS-P35 and UAS-dIAP lines were provided by Dr. David Wassarman at the University of Wisconsin, Madison. The following lines were obtained from the Bloomington Drosophila Stock Center: *string* ^EY12388^(#20349); *greatwall* ^EP515^/Tm6b (#17174); *BobA* ^f03024^(#18609); *Hey* ^f06656^(#18997); *Dl* ^RevF10^ *Ser* ^RX82^(#6300); *E(Spl)* ^rv1^(#199); *Pabp2* ^KG02359^/CyO(#13212); *Pabp2* ^01^/SM5 (#9838); *Rs1* ^K09514^(#10885); *Nup50* ^KG09557^(#15198); Exel6055(#7537); Exel17094(#7859); Exel17095(#7860); Exel16057(#7539); Exel16058(#7540); Exel17096(#7863); DrosDel1735(#9275); DrosDel1742(#9276); DrosDel1725(#8941).

### Western Blot Analysis

Transgenic flies were homogenized in high salt lysis buffer. Proteins were resolved by SDS-PAGE and detected via Western blotting as previously described [34]. TDP-43 was detected using specific antibody from Proteintech (#10782-2-AP).

### Survival Analysis

For survival analysis, flies were aged at 25°C with no more than 25 flies per vial. Vials were changed on a 3–5 day cycle. Death events were scored on a daily basis. Rescue in longevity was defined as greater than 5% increase in median lifespan in addition to the statistical threshold according to the Log-rank (Mantel-Cox) test, p<0.05. In the survival graphs shown, each set of experiments was done in the same time period with the corresponding control subjects in order to control longevity variation caused by environmental factors. Both genders were used in the survival assay unless otherwise specified.

### Statistical Analysis

Statistical analysis was performed using GraphPad Software, San Diego California USA. For survival analysis, Log-rank (Mantel-Cox) test was performed by comparing testing subjects to the D42-Gal4/UAS-TDP-43 or D42-Gal4/+ control. For qPCR analysis, data were presented as mean ±SEM and analyzed by Student’s t-test. The significance of statistical difference was denoted by NS (p>0.05); *(p<0.05); **(p<0.001); ***(p<0.0001).

### Microarray Analysis

To examine relative gene expression, transgenic fly heads were homogenized in Trizol reagent. RNA was then purified using chloroform extraction and ethanol precipitation. Labeling of RNA and hybridization was performed at the UW Gene Expression Center. Nimblegen 4-plex *Drosophila* whole genome microarrays were used, and the data analyzed using Arraystar.

### Quantitative PCR Analysis

Total RNA from 20 fly heads per genotype was extracted by Trizol reagent (Invitrogen) and reverse transcribed by iScript cDNA synthesis kit (BioRad). Quantitative PCR was performed by using iTaq SYBR green supermix (BioRad) according to the manufacturer’s protocol. Real-time PCR data were collected using BioRad CFX96 platform. GAPDH was used as reference gene. Relative mRNA level was calculated by normalizing to the control sample. The following primers were used in our qPCR analysis (forward primer listed first): nACRbeta: ATGCGGCGGACGCAGTACAC, TCGCTGATGCTGTCACGCACAT; Nek2: GCCCTACATCTGGCGGGTGC, TTTGGCATTCCCGGCCGCAT; arrow: TCGACAAGTCGCTGGTGTGCG, CGGCCGAGATGCAGAGCTTGTT; couch potato: ACAGGATCTGCAGCAGGGTGTA, TGGGCTGTGGTTTGGGTTTGCT; three rows: TGCCACGCGTAGTTCCCTGC, TGACAGGCTTCAATGGATCGCTGA; cannonball: TGGGACTCGAAATTGCTTGCTCCG, TCGGTGGGCGATCCATCTGA; BobA: ACTGAGGAGGAGCACCAGAA, ACCTCCAAGGCACACAAGTC; Tom: CAACTCTCAACCGAGTGCAA, GGGTTTGACCAGTTTCCTCA; Hey: TCGGATGAATCGATGACAGA, TAAGATGCAGCAGCAACCAC; Ucp4B: AAATGCAAATGGAGGGACAG, GAACTCGGCAATCAGAAAGC; chickadee: TCACGGCTTGTGTTGTCTTC, AACTGATCAGCGGCTTTGAC; polo: GCGTATGCCCCATTTACACT, GTGCGGAAGTTCTTCTCCTG; string: ATTCCGGTGCGCATTTATAC, GAATAGCGGCTTTCTCGTTG.

### *In vitro* RNA Binding Assay

*In vitro* RNA-binding assays were performed according to Kim et al. with modifications [57]. A 3-kb genomic fragment spanning the entire *Hey* gene locus was subcloned into the NotI/BglII sites of pDS129.36 vector. *In vitro* transcription of the plasmid was carried out as described [57] and different amounts of Biotin-labeled Hey pre-mRNA (0, 1 or 10 pM) were incubated with 0.1 ug of HA-TDP-43 in RNA binding buffer (20 mM HEPES (pH 7.5), 5 mM of MgCl2. 50 mM KCl, 150 mM NaCl, 0.5 mM dithiothreitol, 10% Glycerol) for 20 min at 30°C and then cross linked with 1200 J/m^2^ of UV. Streptavidin beads (Novagen) were added and incubated overnight at 4°C. The beads were washed with an RNA binding buffer and eluted with an SDS loading buffer. Both elute and input fraction was analyzed by SDS-PAGE and Western blotting with anti-HA antibodies (Sigma).

### Immunohistochemistry and Confocal Microscopy Analysis

Fly 3rd instar larval brains were dissected and immunostained as previously described [68]. Ventral nerve cords were stained with primary antibody against TDP-43 (1∶50; Proteintech #10782-2-AP) and nuclear lamin (1∶50; Hybridoma Bank #ADL84.12) for 48 h at 4°C. Alexa-488 conjugated goat anti-rabbit and Alexa-594 conjugated goat anti-mouse secondary antibody were used at 1∶100 for 24 h at 4°C. Images were collected using Olympus FluoView FV1000 Confocal system. Data were processed using ImageJ program.

## Supporting Information

Table S1 (XLS)

Table S2 (XLS)
